# Essay content and style are strongly related to household income and SAT scores: Evidence from 60,000 undergraduate applications

**DOI:** 10.1126/sciadv.abi9031

**Published:** 2021-10-13

**Authors:** AJ Alvero, Sonia Giebel, Ben Gebre-Medhin, anthony lising antonio, Mitchell L. Stevens, Benjamin W. Domingue

**Affiliations:** 1 Stanford University, Stanford, CA, USA.; 2 Mount Holyoke College, South Hadley, MA, USA.

## Abstract

There is substantial evidence of the relationship between household income and achievement on the standardized tests often required for college admissions, yet little comparable inquiry considers the essays typically required of applicants to selective U.S. colleges and universities. We used a corpus of 240,000 admission essays submitted by 60,000 applicants to the University of California in November 2016 to measure relationships between the content of admission essays, self-reported household income, and SAT scores. We quantified essay content using correlated topic modeling and essay style using Linguistic Inquiry and Word Count. We found that essay content and style had stronger correlations to self-reported household income than did SAT scores and that essays explained much of the variance in SAT scores. This analysis shows that essays encode similar information as the SAT and suggests that college admission protocols should attend to how social class is encoded in non-numerical components of applications.

## Teaser

Essay content and style have stronger relationships to household income than do SAT scores and strongly predict SAT scores.

## INTRODUCTION

The information selective colleges and universities (defined by the Carnegie Classification of Institutions of Higher Education as schools that deny admission to at least 20% of applicants) use when evaluating applicants has been a perennial ethical and policy concern in the United States. For a century, admission officers have made use of scores on standardized tests (SAT in particular) to assess and compare applicants. Proponents of standardized tests have argued that they enable universal and unbiased measures of academic aptitude and may have salutary effects on fairness in evaluation when used as universal screens (*1*–*4*); critics have noted the large body of evidence indicating a strong correlation between SAT scores and socioeconomic background, with some having dubbed the SAT a “wealth test” (*5*, *6*). Given the economic and social benefits of a college degree and the increased demand for admissions to selective colleges and universities (*7*), the controversy surrounding the SAT is likely to persist.

There are many other components of admission files, however, including candidates’ primary opportunity to make their case in their own words: admission essays. Yet, there is little comparative literature on the extent to which these materials may or may not covary with other applicant characteristics. How, if at all, do admission essays correlate with household income and SAT scores? Advances in machine learning have made it possible to analyze personal statements and other historically less quantifiable components of admission files at scale.

The movement for test-optional evaluation protocols (*8*, *9*) has gained more momentum in light of the public health risks associated with in-person administration of the SAT and other standardized tests used in college admissions during the COVID-19 (coronavirus disease 2019) pandemic. To the extent that the elimination of standardized tests recalibrates the relative weight of other application materials, the basic terms of holistic review—the current standard of best practice for jointly considering standardized tests alongside qualitative components of applications (*10*–*12*)—require fresh scrutiny. The May 2021 decision by the University of California, a university system serving nearly 300,000 students that is a bellwether for national trends in higher education, to no longer consider standardized test scores compels a thorough reconsideration of the remaining components of admission files and how they relate to applicant characteristics such as socioeconomic status.

**Fig. 1.**
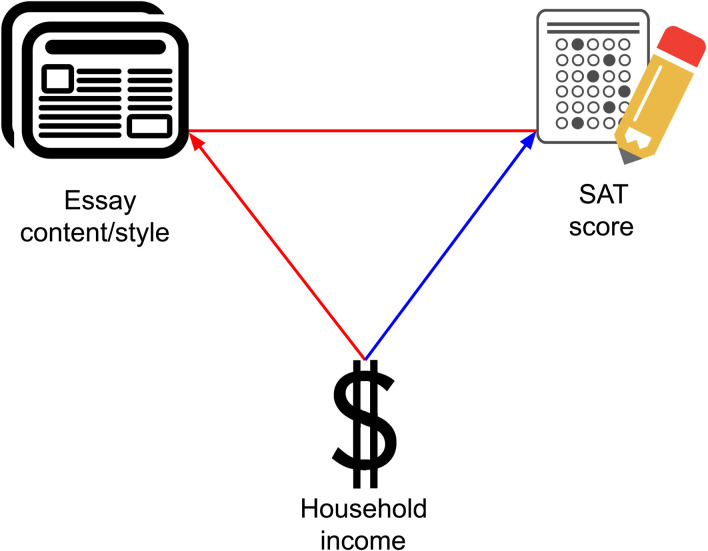
Conceptual model. Visualization of previous work, represented by a blue line, and our study, represented by red lines, on the relationship between application materials and household income.

To help inform this national conversation, we analyzed a dataset comprising information from 60,000 applications submitted to the nine-campus University of California system in November 2016 (for admission to the 2017–2018 academic year) with the goal of observing the relationship between essay content and style, self-reported household income, and SAT score. The basic conceptual model that we tested is shown in Fig. 1. The well-known fact that SAT scores show associations with household income (*5*, *6*) is captured by the blue arrow. We find such an association in our dataset as well. Our primary aim was to test relationships along the red lines.

To do so, we juxtaposed results from an unsupervised, probabilistic approach using correlated topic modeling (CTM) (*13*, *14*) with results from a dictionary-driven analysis using proprietary software Linguistic Inquiry and Word Count (LIWC) (*15*). We chose these two techniques because they are commonly used for analysis of textual data in other evaluative contexts (*16*–*18*). Prior research using computational readings, where textual data are analyzed using computational and statistical methods, has considered the relationship between admission essay vocabulary and grammar with author gender, household income, or subsequent academic grades (*19*–*22*); we extend this emerging literature by comparing the content and style of undergraduate admission essays, household income, and standardized test scores at scale. In so doing, we focused on the relationships among the materials that a student submitted in the application, not the relationships between those materials and the admission decision and/or essay rating. However, it is important to note that previous studies on the relationship between income and SAT scores have similarly considered preadmission data (*5*, *6*). Here, we similarly focus on the data (essays) provided by and associated with applicants rather than university decision-making processes.

Our analysis proceeded as follows. First, we identified patterns in the dictionary features (LIWC) and the topics (CTM) that emerged from computational readings of the essay corpus. We refer to the CTM-generated features as essay “content” and the LIWC-generated features as essay “style.” These metrics may not resemble those used in the evaluative process undertaken by human readers (i.e., admission officers), but our objective was to reveal patterns in the application materials rather than to speak to how essays are assessed during the admission process. Second, we used these features to examine patterning of essay content and style across household incomes. We found that essay content and style had stronger correlations with household income than did SAT score with household income. Third, we identified strong associations between SAT score and essay content and style. These associations persisted even when we stratified our analyses by household income, indicating that they are not due purely to the well-known income-SAT association. Together, these findings suggest that many of the associations with socioeconomic status deemed concerning with regard to using SAT scores in college admissions also pertain to admission essays. Findings are of immediate policy relevance given the changes in evaluation protocols that will come if standardized test scores are to be eliminated from college applications, a growing trend nationwide and a reality for the next round of University of California admissions.

## RESULTS

### Describing essay content and style via dictionary features and probabilistic topics

In the application cycle considered here, applicants to the University of California were given eight essay prompts and were required to write responses to any four prompts. We focused our analysis on a random sample of *n* = 59,723 applicants for first-year admission. Additional information about the sample can be found in Materials and Methods. As each applicant wrote four essays, our corpus consisted of 238,892 essays. Each essay was limited to 350 words (the average essay contained 348 words); applicants submitted 1395 words on average across the four essays. We describe results based on analysis of what we call the “merged” essay: each applicant’s four essays merged into a single document. In the Supplementary Materials, we discuss the analysis of essays written to two specific prompts; results were similar and appear in tables S1 and S2.

We captured essay content via CTM and essay style via LIWC. These approaches are distinctive in their foci: Content denotes what applicants wrote about in their essays while style denotes how applicants deployed language in their essays. We separately describe each approach.

### Essay content

CTM (*13*) is a probabilistic, data-driven strategy that relies only on the words in the essays (i.e., it uses no external data). Topic modeling identifies semantic content (i.e., meaning) via a generative, probabilistic model of word co-occurrences. Words that frequently co-occur are grouped together into topics and usually show semantic cohesion (e.g., a given topic may include terms such as “baseball,” “bat,” and “glove” because these words tend to co-occur in a document). A document is assumed to consist of a mixture of topics; CTM analysis involves first specifying the number of topics in a corpus and then estimating the mixture proportions of topics for each document in the corpus. Topic modeling has been used to measure changes in research publication topics and themes over time in academic fields such as statistics and education (*18*, *23*, *24*); it has also been used for more focused studies such as measuring the relationship between seller descriptions and sales in an online marketplace (*25*). For a comprehensive overview of topic modeling, see (*26*).

Via topic modeling, we generated 70 topics across the full corpus that we used as independent variables for analysis. Details regarding topic construction, including explanations for the “highest probability” and “frequent exclusive” metrics used to identify the words most representative of a given topic, can be found in Materials and Methods. Merged essay topics included a wide variety of themes (e.g., winning competitions, social anxiety, medical experiences, and language experiences; see table S3) and topics related to specific majors (e.g., physics, computer science, and economics).

We observed a range of associations between themes and either household income or SAT score. For example, essays with more content on “Human Nature” and “Seeking Answers” tended to be written by applicants with higher SAT scores (*r =* 0.53 and *r =* 0.57, respectively); in contrast, essays with more content about “Time Management” and “Family Relationships” tended to be written by applicants with lower SAT scores (*r* = −0.40 and *r* = −0.26, respectively). Table 1 shows the five topics most positively correlated with income (in blue) and the five most negatively correlated with household income (in red), along with excerpts from the essays with the highest estimated proportion of the topic (i.e., we offer excerpts from the essay most representative of the topic). A full visualization of topic correlations with household income and SAT score can be found in fig. S1 and a full list of topics in table S3.

**Table 1.** Topics most positively (blue) and negatively (red) correlated with household income and SAT score along with excerpts from essays with highest topic score.

**Merged essay topic**	**Highest probability words**	**Frequent exclusive words**	**Excerpt from essay with highest** **topic score**
Seeking answers (income *r* = 0.28; SAT *r* = 0.57)	question, book, like, research, read, answer, ask	telescop, astronom, map, probe, column, constel, encyclopedia	“Ever since the big bang took place, particles have been hovering around the universe for billions of years. Some of them now constituted me, but who knows where they were billions of years ago? Why couldn’t they have come from Mars?”
Human nature (income *r* = 0.21; SAT *r* = 0.53)	world, human, natur, passion, beyond, complex, explor	inher, manifest, notion, philosophi, nuanc, facet, myriad	“From a young age, I have found a fascination in the art of rhetoric and its influence on humanity…I believe as cognitively complex individuals we should maximize our ability as a collective species to understand the very nature of our surrounding”
China (income *r* = 0.29; SAT *r* = 0.42)	chines, studi, student, also, time, china, school	china, provinc, hong, kong, chines, shanghai, wechat	“I served as the Chunhui emissary and participated in the voluntary activities in the ‘Chunhui Action’ in Qixingguan District in Bijie City in Guizhou province. Our team went to the povertystricken area in Qixingguan District and helped build”
Achievement words (income *r* = 0.12; SAT *r* = 0.39)	result, provid, initi, began, becam, academ, effort	dilig, remain, util, attain, endeavor, initi, simultan	“Rather than taking the fundamental classes to proceed through high school, I chose to additionally push myself out of comfortability and undertake the strenuous task of taking Advanced Placement classes. Prior to entering my senior year, I have successfully passed a total of two honors and five Advanced Placement courses, all while managing both extracurricular activities and favorable pastimes”
Despite words (income *r* = 0.05; SAT *r* = 0.32)	howev, one, may, rather, even, simpli, fact	simpli, rather, may, fact, truli, consid, howev	“To this day, I cannot begin such an ambitious project, though perhaps that is simply because it is so enterprising. Perhaps if the attempt was made to write something shorter and more reasonable I could have succeeded, could have written something to be remembered. But I never did, though I still have the chance. Maybe I will. Maybe today will be the day I decide to write”
Time management (income *r* = −0.23; SAT *r* = −0.40)	time, work, help, get, school, abl, go	homework, manag, get, stress, done, stay, procrastin	“I do try hard to make sure that I complete my assignments by a certain time, but sometimes I have to stay up later than I expected to make sure I finish everything. This has affected my achievement by making me have to focus on one class more than the other. This has proven to be a big challenge, but I plan to overcome it”
Helping others (income *r* = −0.14; SAT *r* = −0.34)	peopl, help, can, make, way, differ, other	peopl, can, other, someon, everyon, differ, way	“When I am helping the students, I have to take charge, show them how each step is done to help them complete whatever it is that they are doing. If I see one of them is having trouble, then it is my duty as a leader to show them how to do it so they will understand how to do it the next time. Being a teacher’s assistant is a hard job but it gives me responsibility skills that I will need in the future”
Tutoring groups (income *r* = −0.25; SAT *r* = −0.42)	help, tutor, colleg, avid, also, go, need	avid, tutor, ffa, et, ag, via, tutori	“It has also taught me to seek help from tutors something that trained me to improve my homework and test taking abilities. Taking advantage of these educational opportunities made me feel empowered and grateful. Through these programs, I had the opportunity to learn valuable skills and tools to ease my transition and help me be successful in a four year college environment”
Preference words (income *r* = −0.13; SAT *r* = −0.32)	also, like, thing, realli, subject, lot, alway	realli, lot, thing, good, favorit, influenc, enjoy	“My greatest talent or skill is acting. I absolutely love acting, and it is one of my greatest talents. Just recently I took an acting class and it is one of the best decisions I have ever made…My favorite monologue that I performed in my class was Charlie and the Chocolate Factory, it really matched me”
Education opportunity (income *r* = −0.21; SAT *r* = −0.29)	colleg, educ, opportun, take, advantag, attend, school	advantag, educ, colleg, opportun, credit, graduat, prep	“I also took real college classes with other college students which are transferable if accepted by other colleges or universities. While it has been a major educational opportunity, it has also been a educational barrier which I have had to overcome. The more advanced high school education and full college courses have required me to put a large amount of my time and effort into my education”

### Essay style

LIWC (*27*) relies upon an external “dictionary” that identifies linguistic, affective, perceptual, and other quantifiable categories that model patterns of writing style. LIWC generates 90 such features [described by LIWC developers as “categories” of writing (*28*); we call them “dictionary features” throughout this paper) based on word or character matches across a given document and the external dictionary. These include simple word and punctuation counts, grammatical categories such as pronouns and verbs, sentiment analysis, specific vocabularies such as family or health words, and stylistic measures such as “narrative writing.” LIWC also generates composite variables from combinations of categories, such as analytical writing, based on the frequency of function words such as articles and prepositions. For example, sentences using more personal pronouns such as I, you, and she score lower in the analytical category than do sentences using more articles such as a, an, and the. We chose LIWC because it has been widely used and is well understood in the social sciences (*16*–*18*). It has seen extensive methodological validation (*16*, *17*, *22*, *28*, *29*) and has been used to analyze a different corpus of college admission essays (*14*). Our models used 89 of the LIWC categories as independent variables. (See Materials and Methods for additional details.)

As we observed in our CTM analysis, there was a range of associations between LIWC dictionary features and either household income or SAT score. Counts of total punctuation (*r* = 0.34), comma use (*r* = 0.434), and words longer than six letters (*r* = 0.38) were positively associated with SAT score, for example, while function words (e.g., prepositions and articles; *r* = −0.42) and verbs (*r* = −0.47) were negatively associated with SAT. Correlations for household income followed a similar pattern. These findings parallel prior work focusing on a smaller sample of admission essays submitted to a single institution (*21*).

### Complementarity of essay content and style

**Fig. 2.**
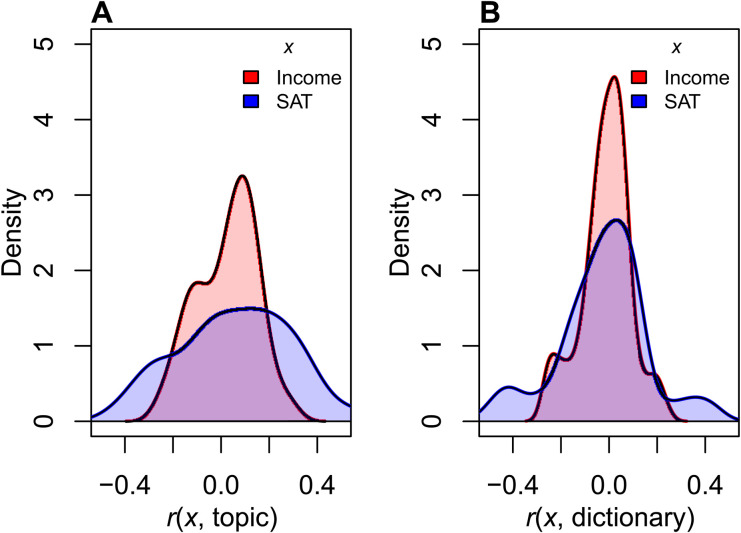
Densities of correlations of essay content and style with SAT scores and household income. (**A**) By topics and (**B**) by dictionary features.

Both methods for quantifying essays produced features that showed varying levels of association with household income and SAT score (see Fig. 2). As CTM and LIWC have important conceptual and methodological differences, we view them as complementary in that they allow us to test whether multiple potential techniques may yield quantified essay features similarly patterned across household income and SAT score. The relatively weak correlation between topics and dictionary features within an essay (average correlation for topics and dictionary features for merged essays: *r* = 0.001; median correlation: *r* = 0.011) further suggests that the methods are complementary rather than redundant.

In the following analyses, we probe the relative magnitudes of the associations in Fig. 1. The fact that many specific correlations of individual features are relatively large (see Fig. 2 and fig. S1) anticipates strong patterning of essay content and style (across all features) of household income and SAT score.

### Essay content and style were more strongly associated with household income than with SAT score

Having developed quantitative representations of both essay content and style, we then estimated the strength of the relationships between the types of essay features, household income, and SAT score. We first treated household income as the dependent variable. We compared adjusted *R*^2^ from three out-of-sample linear regression models: Model A used SAT scores as a predictor [SAT Evidence-Based Reading and Writing (EBRW) and SAT Math were tested separately]; models B and C used topics and dictionary features, respectively, as predictors. In Fig. 1, model A represents the blue arrow while models B and C represent the red arrow between household income and essay content/style. Essays written by applicants who reported household income below $10,000 (*n* = 1911) were included in the topic modeling but excluded from the regression analyses because we suspected that many of those applicants may have misreported parental income (*19*) (final sample *n* = 57,812). Note that models B and C use essay content and style as predictors rather than as dependent variables; compressing the essays into a single outcome variable would have resulted in substantial information loss.

As shown in Table 2, between 8 and 12% of variation in household income is explained by SAT score. These estimates are comparable to those of previous work; data from seven University of California campuses collected between 1996 and 1999 yielded similar associations [e.g., *R*^2^ ≈ 0.11 between logged income and total SAT score; see table 1 in (*29*)]. Somewhat more variation is explained by Math scores than by EBRW scores, and the total SAT score is roughly as predictive as the Math score alone.

**Table 2.** Out-of-sample prediction error for prediction of household income by topics, dictionary features, and SAT scores using 10-fold cross-validation (CV).

**Model**	***R* ^2^**	**95% confidence** **interval**
A. SAT predicting household income		
SAT Composite	0.119	[0.115, 0.124]
SAT EBRW	0.083	[0.079, 0.087]
SAT Math	0.120	[0.115, 0.124]
B. Topics predicting household income		
Topics	0.161	[0.157, 0.167]
C. Dictionary predicting household income		
LIWC	0.129	[0.127, 0.136]

We found that essay content and style (i.e., models B and C) were each more predictive of household income than SAT score. Topics (*R*^2^ = 16%) were better predictors of household income than were dictionary features (*R*^2^ = 13%). Note that topics showed higher predictive performance despite the fact that the dictionary feature–based model used 19 more predictors and external data.

Results for prompt-specific essays (i.e., one of the four essays an applicant wrote), shown in the Supplementary Materials (tables S1 and S2), were somewhat weaker, suggesting that some degree of respondents’ selection of prompts and/or the university’s prompt-specific language could have played a role in forming the primary associations on which we focused here. It is also possible that the difference in predictive performance was simply due to the merged essays providing more data than the individual essays in terms of word count and sample size.

As a contrast, we also considered five readability metrics commonly used in education research (*30*–*34*) in place of our primary metrics of essay content and style. We found much weaker associations between readability and SAT score (*R*^2^ < 0.1; see table S4), indicating that our essay content and style metrics are more closely related to socioeconomic status and traditional markers of academic performance than other widely used measures of text.

Given longstanding concerns about the strength of the relationship between SAT score and socioeconomic status, our finding that a similar relationship exists between essay content (topics) and style (dictionary features) and socioeconomic status is noteworthy. We further discuss implications in Discussion and turn now to a consideration of associations between SAT score and essay content and style.

### Essay content and style strongly predicted SAT score

We next focused on the interplay between SAT scores and essay content and style. Specifically, we assessed whether essay content and style can explain variation in applicant SAT scores. Table 3 summarizes the relationship that we observed between essay content and style and SAT score. Prediction of SAT score was robust: Approximately 43 to 49% of total SAT score were explained by essay content and style, with some variation around these values for SAT EBRW and SAT Math. The root mean square error (RMSE) of our models for the total SAT score was 124.87 (topics) and 130.85 (dictionary features).

**Table 3.** Out-of-sample prediction error for prediction of SAT score by topics using 10-fold CV.

**Essay**	***R* ^2^**	**95%** **confidence** **interval**	**RMSE**
Topics			
SAT Composite	0.486	[0.478, 0.489]	124.87
SAT EBRW	0.428	[0.419, 0.431]	64.83
SAT Math	0.473	[0.466, 0.477]	74.34
Dictionary			
SAT Composite	0.436	[0.428, 0.440]	130.85
SAT EBRW	0.369	[0.362, 0.374]	68.05
SAT Math	0.405	[0.399, 0.410]	78.96

On the basis of this finding, we argue that essay content and style are far more predictive of SAT score than, for example, high school grade point average (GPA) [*R*^2^ = 0.04 between high school GPA and total SAT score (*29*), although those results were based on an older version of the SAT and might not be fully comparable with the results reported here]. Our findings are especially noteworthy given that topics and dictionary features were generated in an a theoretical manner that was blind to information about the applicants’ family background or academic performance, pointing to potential complications in requiring essays as pieces of “nonacademic” information about applicants.

Collectively, findings from Tables 2 and 3 suggest that essay content and style—themes, diction, grammar, and punctuation—encode substantial information about family background (as captured by household income) and academic performance (as captured by the SAT). Similarly, prediction results from a model that used both content and style essay features were higher than models described herein that focused separately on essay content or style (*R*^2^ = 0.17 for household income and *R*^2^ = 0.53 for SAT; see table S5). Designers of application protocols that include essays will need to take the strength of the relationships among essay content and style, family background, and academic performance into consideration, a topic we return to in Discussion.

### Associations between essay content and style and SAT score persist within household income decile

We have shown that essay content and style are associated with SAT score; this relationship may be partially due to the fact that both SAT score and essay content and style are associated with income. To study whether the relationship between essay content and style and SAT persisted after controlling for socioeconomic status, we split our data by household income decile and then repeated our test from Table 3 within each income decile. This approach, modeled after a related study (*20*), was designed to determine whether the observed patterns in Table 3 had a root cause—socioeconomic status, see Fig. 1—or whether there were distinctive relationships between essay content and style and socioeconomic status that predicted SAT score.

**Fig. 3.**
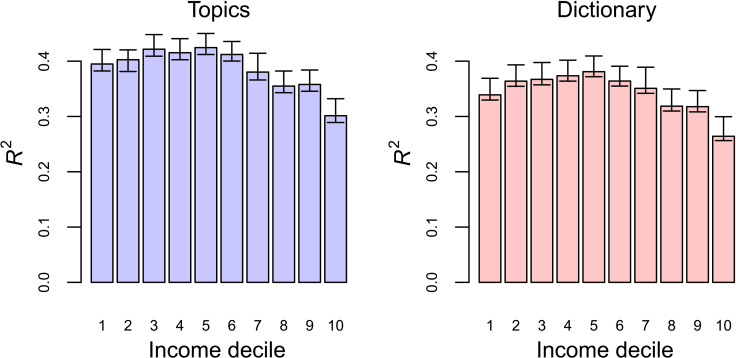
*R*^2^ of total SAT when stratified by household income decile. Explained by topics (**left**) and dictionary features (**right**).

After stratifying our data by household income decile, we found that essay content and style remained predictive of SAT score (see Fig. 3). Essays written by applicants in the highest household income deciles had the weakest relationship with SAT score. This was true for both style and content: Associations were between *R*^2^ = 0.25 and *R*^2^ = 0.30 for the highest income applicants. We observed the strongest relationship between essay topics and SAT score for middle-income students: *R*^2^ = 0.40. One potential explanation is that observable variation in SAT score is smallest in the highest deciles of household income (see table S6), suggesting that the variation illustrated in Table 3 is not purely a signature of household income.

## DISCUSSION

The use of standardized test scores in selective college admissions, long a controversial strategy, is being reconsidered by many institutions. Major changes to admission processes, such as discarding SAT scores, require an evidence-based reconsideration of how the remaining components of application files relate to applicant demographics. We analyzed the relationships between applicants’ self-reported household income, SAT score, and essay content and style from a random sample of 240,000 essays submitted by 60,000 applicants to the University of California in November 2016. We found that essay content and style were more strongly associated with household income than was SAT score. We also found that essay content and style are strong predictors of applicant SAT score, with *R*^2^ of nearly 50% in some models. The relationship between essay content and style and SAT score was strongest for middle-income students and weakest for high-income students. The associations reported here should inform ongoing discussion about fairness, bias, and transparency in holistic review during the admission process by providing insight about the extent to which qualitative components of applications bear the signature of applicants’ family backgrounds.

Our results confirm previous research illustrating that socioeconomic status is correlated with tests such as the SAT and extend such research to suggest that class markers are present in aspects of the admission file that are often perceived as qualitative counterweights to standardized assessments. Standardized tests are designed to produce a concise ranking among applicants (although, there, it is becoming increasingly common for test scores to be interpreted alongside contextual markers such as socioeconomic status and average test scores at an applicant’s school; see, for example, https://secure-media.collegeboard.org/pdf/environmental-context-dashboard-faqs.pdf); by contrast, essays have no inherent hierarchical relation with each other and instead provide readers with contextual and noncognitive information for evaluating applicants (*35*). Essays are intended to provide information about an applicant’s resources, conditions for learning, and personal characteristics such as motivation, resilience, leadership, and self-confidence. The expressed purpose of admission essays, and of holistic review more generally, is to enable consideration of applicant attributes beyond what is captured in a few easily comparable numbers (*12*, *36*–*38*).

Yet, however its constituent parts are conceptualized, the entire evaluation process is ultimately an effort to sort applicants along a single dimension: accept or reject. While it may not be anyone’s intention to rank admission essays, they are, nevertheless, one component of a process that converts applicant fitness against a binary outcome. In theory, essays allow applicants to present their case for admission through idiosyncratic narratives, which, in turn, help admission officers consider the entire profile of applicants in trying to construct a class that fulfills multiple, and sometimes competing, institutional priorities (*12*). Our findings suggest that such holistic review may be redundant in an unanticipated way: Household income, standardized test scores, and essay content and style are highly interrelated.

The present study cannot speak to how admission officers evaluate essays or about the current role of essays in holistic review more generally. Addressing these matters will require dispassionate, up-close observation of how admission officers make decisions, as Stevens did 20 years ago (*12*). His ethnographic work indicated that essays had a negligible role in final decisions, but this may be different in the more competitive contemporary context and in the wake of test-optional policies. Future studies might combine qualitative and quantitative observational strategies to understand whether and how admission officers’ readings of essays vary with the family backgrounds of applicants. While such work remains to be done, our findings about the interrelatedness of household income, standardized test scores, and essay content and style can inform current public discourse about the meaning and value of the different kinds of information comprising college applications. In short, our work indicates that merely eliminating SAT scores from consideration in no way eliminates the signature of class from application materials.

Concerns about associations between socioeconomic status and SAT score should therefore be expanded to include what have been long understood as “qualitative” components of applications but are now amenable to computational reading at scale. If analysis consistently finds that essay content and style reflect socioeconomic resources in nontrivial ways, then essay requirements may require the same level of critical scrutiny that standardized testing has been receiving. Removing SAT scores from admission files would likely remove practical barriers to selective colleges for at least some students (*39*) [removing SAT scores may also limit applicants’ ability to know how they might fare in college, a crucial signal (*2*)], but if essays encode as much information as SAT scores and have a stronger relationship with household income, then the use of essays in admission decisions warrants careful consideration by researchers and admission professionals alike.

While there is evidence supporting a relationship between noncognitive attributes and educational outcomes in college (*40*), there is, at present, only minimal research on the evaluative content of admission essays. These texts may prove to be a complex mosaic of socioeconomic status, academic ability, educational performance, social context, and individual-level characteristics. Researchers might more closely examine the metrical features of admission essays and extend similar lines of inquiry to other qualitative application components, such as letters of recommendation and interview write-ups. Further, allowing machines to “read” essays either alongside or in place of human reviewers may seem far-fetched to some, but it is standard practice in other settings in education (*41*), and the development of automated protocols for evaluation of candidates in related spaces is no longer hypothetical (*42*).

Applicant essays are not the only prose elements that have the kinds of characteristics described here. Personal statements required for applications to graduate/professional school and by potential employers, even written attempts to secure housing, may display structures as a function of the writer’s socioeconomic background. Our findings dovetail with others from literature in sociolinguistics that emphasizes how language is structured by class (*43*–*45*). We would interpret such structure as a reflection of both an applicant’s life experience presented as personal narrative and language socialization (*46*) [cf. more deficit-oriented interpretations (*47*)]. The applicants were writing to the same prompts with the same submission deadline, and the sociolinguistic variation in their essays does not indicate “better” or “worse” language. There is nothing inherently better about writing more about seeking answers rather than time management or frequently using longer words and commas. Essay ratings and admission decisions would also not indicate better or worse language, but rather the preferences of the University of California with respect to a specific applicant. While computational tools will undoubtedly be of use in future studies of personal statements, as well as the practice of assessing them, this sociolinguistic lens (*48*–*49*) may also be a valuable analytic perspective for framing the results of these studies.

Ever more fierce competition for limited seats at prestigious schools will require constant attention to ensure any degree of fairness in evaluation protocols. Campbell’s law—“the more any quantitative social indicator is used for social decision-making, the more subject it will be to corruption pressures and the more apt it will be to distort and corrupt the social processes it is intended to monitor” (*50*)—suggests that there are no simple means of ensuring fairness. Elimination of standardized tests will not increase the number of seats at elite schools, but it may increase the number of applications that those schools receive. We suspect that it will be increasingly tempting for admission offices to pursue automated means of reviewing application portfolios; doing so would almost inevitably incite college hopefuls, especially the most savvy, to devise new ways of presenting their applications in the most flattering light. Whatever the future of holistic review, our results strongly suggest that the imprint of social class will be found in even the fuzziest of application materials.

## MATERIALS AND METHODS

### Data

Our data, provided by the University of California, was a random sample of 60,000 anonymized applications drawn from an application pool of more than 165,000 individuals who submitted application materials in November 2016 for matriculation in fall 2017. The shared data included applicant essays, raw household income, SAT scores, and various personal characteristics about each applicant. The essays were required components of applications. Each applicant was expected to write four essay prompts out of eight total choices, yielding a dataset of 240,000 essays. Before any analysis, we removed all applicants who wrote essays for the transfer admission prompt and applicants with merged essays shorter than 50 characters (*n* = 59,723). The prompts are listed in the Supplementary Materials and described in more detail in an ongoing work (see https://osf.io/preprints/socarxiv/njhg9).

### Text preprocessing

We focused largely on merged essays: the text resulting from collapsing all four admission essays written by each applicant into a single document. We preprocessed these documents before analysis using the quanteda package in R (*51*) (see also the Supplementary Materials). We removed English stop words (from quanteda’s built-in list), stemmed the words using the Porter Snowball stemmer (*52*), lowercased all characters, and removed all punctuation and numbers. We also ensured that there was a white space character after all periods and commas (we found that many students did not add expected spaces after periods and commas). For example, some applicants might have written “This is a sentence.This is a different sentence.” rather than “This is a sentence. This is a different sentence.”

### Hyperparameter tuning

**Fig. 4.**
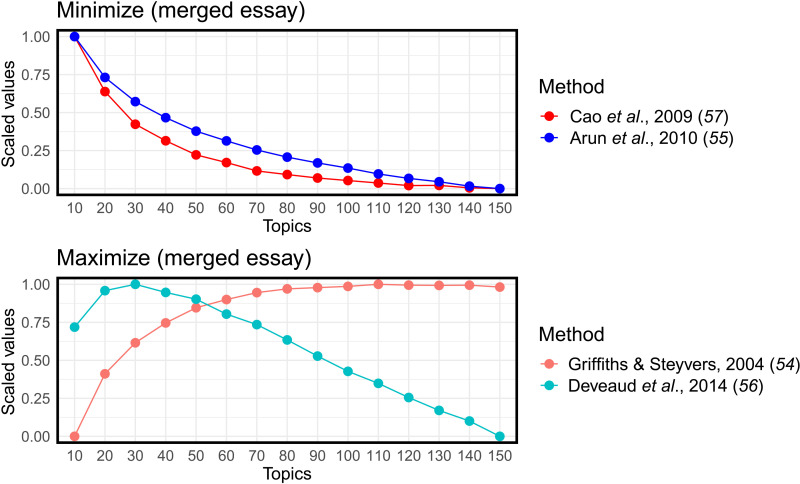
Results from ldatuning suggesting 70 topics. (**Top**) Algorithms that suggest number of topics based on minimizing certain statistical properties of the data and model and (**bottom**) algorithms that suggest number of topics based on maximizing certain statistical properties. The number of topics where the four algorithms are closest (70) is chosen for our model.

Hyperparameter tuning for topic modeling is a well-known methodological challenge. Because we used the topics as predictors and were less concerned with their semantic coherence and the clarity of the resulting topics, we relied on quantitative measures of topic quality using the ldatuning package in R (*53*) (see also the Supplementary Materials). This package uses four metrics (*54*–*57*) to estimate a reasonable number of topics. Models were tested for 10 to 150 topics, increasing by 10 for each test. After standardizing the results for the four equations, we selected the number of topics that had the best average performance across the four metrics (70 topics for the merged essays and 50 topics for the single essays). See Fig. 4 and figs. S2 and S3 for a visual representation of this approach.

We then used the stm (structural topic modeling) package in R (*14*) (see also the Supplementary Materials) to generate the number of topics suggested by our ldatuning approach. The stm function in the package defaults to CTM when covariates are omitted.

### Reporting top terms for topics

We use the highest probability and frequent exclusive (abbreviated as “FREX”), standard choices for topic identification that are implemented in the software used here (*14*). Additional details are presented in the “Metrics for identifications of top terms” subsection in the Supplementary Materials.

### Dictionary features

We used all the LIWC dictionary features available except for “Dash” because of incompatible formatting between the essays and the dashes detected by LIWC. This generated 89 of 90 possible categories for each essay.

### Linear model details

*R*^2^ estimates for out-of-sample predictions were based on 10-fold cross-validation with a train/test split of 90%/10% to prevent overfitting (*58*). We report the average *R*^2^ across all folds. The 95% confidence intervals were constructed via 10,000 bootstrap replications. RMSE is the root mean square error, the SD of the prediction errors in a model. Given that a single document is approximated as a mixture of topics, the topic scores always sum to unity within an essay. To address collinearity, we removed one topic from model B.

To calibrate our approach, we applied our analytic pipeline to data from a previous study of admission essays (*21*). That previous study uses the LIWC variables from the 2007 version of the software for each applicant’s essay and their SAT equivalent score (many applicants took the ACT). When we use that study’s data in our analytic pipeline, we explain less variation in SAT scores via LIWC variables (*R*^2^ = 0.21) than in our data. This is presumably due to two sampling factors that narrowed the range of content in those essays: The prior study’s data came from students who were admitted to, and eventually enrolled at, a single-campus flagship state institution (University of Texas at Austin), while ours include essays from all applicants of the multicampus University of California. Their study also used a different, older version of LIWC.

## Supplementary Materials

### This PDF file includes:

Supplementary Text Figs. S1 to S3 Tables S1 to S6 Data S1 References
