# Supplementary material for: Essay content and style are strongly related to household income and SAT scores: Evidence from 60,000 undergraduate applications
Source: Sci Adv. 2021 Oct 13;7(42):eabi9031. doi: 10.1126/sciadv.abi9031 (PMC8514086; doi:10.1126/sciadv.abi9031)
Supplement: Supplementary file 1 — Supplementary Text Figs. S1 to S3 Tables S1 to S6 Data S1 References [file sciadv.abi9031_sm.pdf]

Science Advances
AAAS

Supplementary Materials for

Essay content and style are strongly related to household income and SAT scores: Evidence from 60,000 undergraduate applications

AJ Alvero*, Sonia Giebel, Ben Gebre-Medhin, anthony lising antonio, Mitchell L. Stevens, Benjamin W. Domingue*

*Corresponding author. Email: ajalvero@stanford.edu (A.A.); ben.domingue@gmail.com (B.W.D.)

Published 13 October 2021, *Sci. Adv.* **7**, eabi9031 (2021)
DOI: 10.1126/sciadv.abi9031

This PDF file includes:

Supplementary Text
Figs. S1 to S3
Tables S1 to S6
Data S1
References

Supplementary Text

Correlations of essay content with household income and SAT score

Figure S1 shows correlations between dictionary features (left) and individual topics (right); household income and SAT score. The correlations for each feature/topic are ordered by the sum of their respective correlations with SAT score and income (i.e. correlation between feature/topic and income, SAT score, and individual test scores). The most positively correlated features/topics are at the top of the figure and the darkest red color; the most negatively correlated features/topics are the bottom of the figure and the darkest blue color.

Choosing *k* topics

The ldatuning package in R uses four metrics to calculate an optimal number of topics (54–57). See https://github.com/nikita-moor/ldatuning/blob/master/R/main.R. Figures S2 (significant challenge, prompt 5) and S3 (creative side, prompt 2) show results for each method from the ldatuning package, rescaled with mean zero (labeled "Scaled Values" for the y-axes in Figures 4, S2, and S3) in order to make them visually comparable. The optimal value identified by the ldatuning package is the number of topics where each of the four points cluster the closest together, in this case 50. This result was used to choose the number of topics for the models we then used in our analysis of essay content, income, and SAT score.

Metrics for identification of top terms

The stm package in R provides the top terms for each generated topic based on calculations from various metrics. See https://cran.r-project.org/web/packages/stm/index.html. We focus on two metrics: "frequent exclusive" words (which we denote as FREX in equations) and "highest probability" words.

The "frequent exclusive" metric is defined as the weighted harmonic mean of a given word in terms of its overall frequency in the essay corpus and its exclusivity to a given topic (59). This metric, therefore, was designed to balance the *frequency* of a given word and its *exclusivity* to a given topic, relative to other topics. For word f in topic k, $FREX_{fk}$ is the harmonic mean of the word's exclusivity to the topic $\Phi_{f.,k}$ and topic specific frequency $\mu_{f.,k}$:

$$FREX_{f,k} = \left(\frac{w}{ECDF_{\phi_{.,k}}(\phi_{f,k})} + \frac{1-w}{ECDF_{\mu_{.,k}}(\mu_{f,k})} \right)^{-1}$$

"Highest probability" words are those with the highest word-to-topic distribution parameters.

Significant challenge and creative prompts

Tables S1 and S2 show results in parallel to Tables 1 and 2 of the main text but using only essays responding to the "significant challenge" (n = 36,573) and "creative side" (n = 22,768) prompts.

Top terms for merged essay topics

Table S3 lists the top terms for each topic identified via CTM. We created the topic labels based on themes suggested by the top terms.

SAT score distributions: Mean and variance by household income decile

Table S4 shows mean and *SD* of total SAT score as a function of household income. Note that variance is lower in the top household income deciles.

Readability

Table S5 shows associations between readability metrics and household income or SAT score. We derived the readability metrics with the quanteda package in R using the textstat_readability function [51]. See https://cran.r-project.org/web/packages/quanteda/index.html.

Essay prompts

See https://admission.universityofcalifornia.edu/_assets/files/how-to-apply/uc-personal-questions-guide-freshman.pdf for more details.

1. Describe an example of your leadership experience in which you have positively influenced others, helped resolve disputes, or contributed to group efforts over time.
2. Every person has a creative side, and it can be expressed in many ways: problem solving, original and innovative thinking, and artistically, to name a few. Describe how you express your creative side.
3. What would you say is your greatest talent or skill? How have you developed and demonstrated that talent over time?
4. Describe how you have taken advantage of a significant educational opportunity or worked to overcome an educational barrier you have faced.
5. Describe the most significant challenge you have faced and the steps you have taken to overcome this challenge. How has this challenge affected your academic achievement?
6. Think about an academic subject that inspires you. Describe how you have furthered this interest inside and/or outside of the classroom.
7. What have you done to make your school or your community a better place?
8. Beyond what has already been shared in your application, what do you believe makes you stand out as a strong candidate for admission to the University of California?

Figure S1. Correlations, Merged Essays

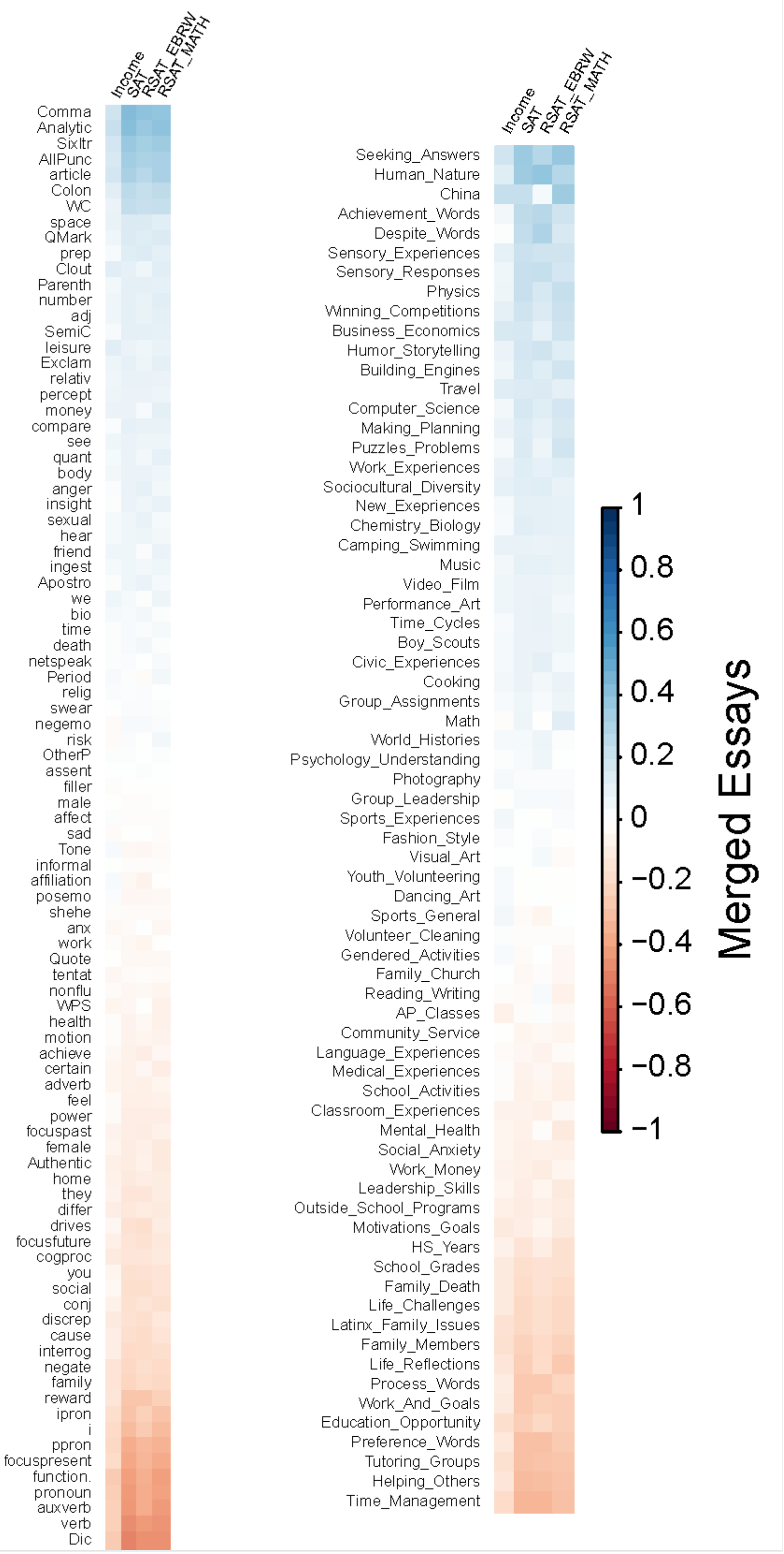

Figure S2. Results from ldatuning Suggesting 50 Topics for Modeling "Significant Challenge" Essays

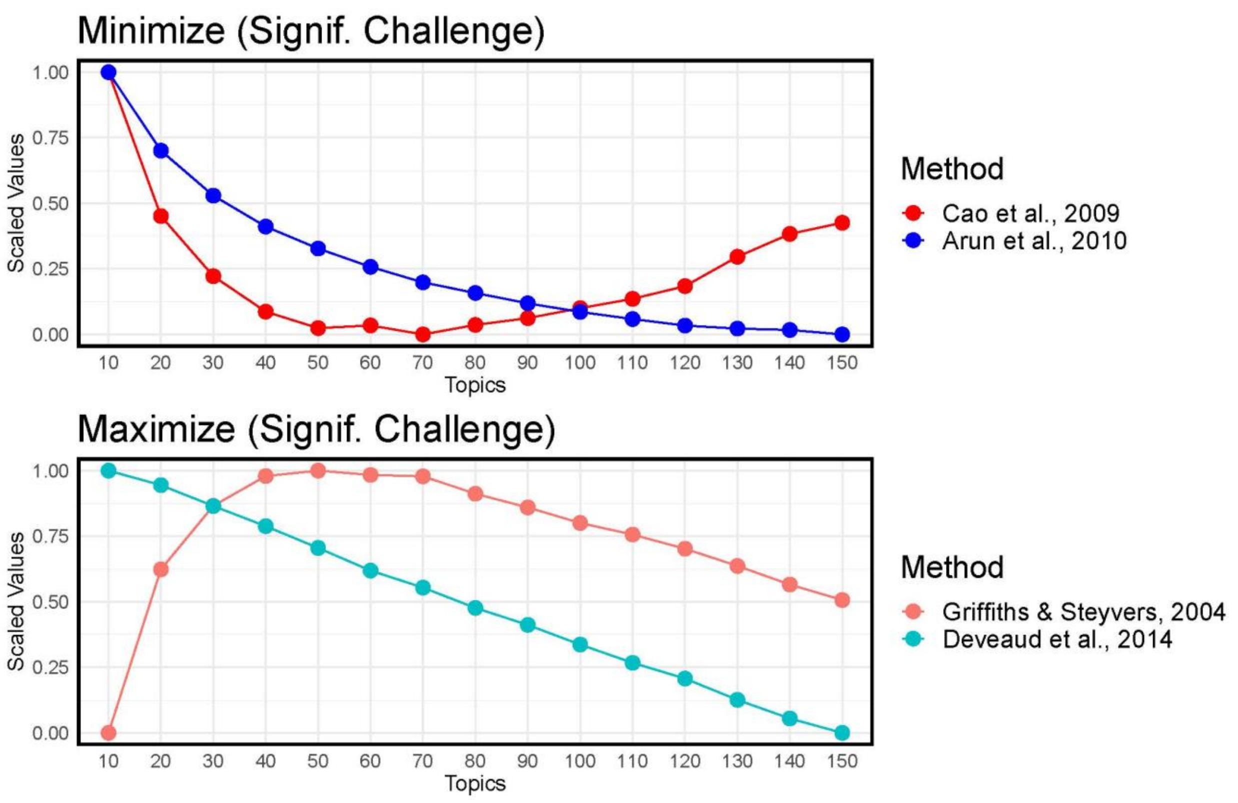

Figure S3. Results from ldatuning Suggesting 50 Topics for Modeling "Creative Side" Essays

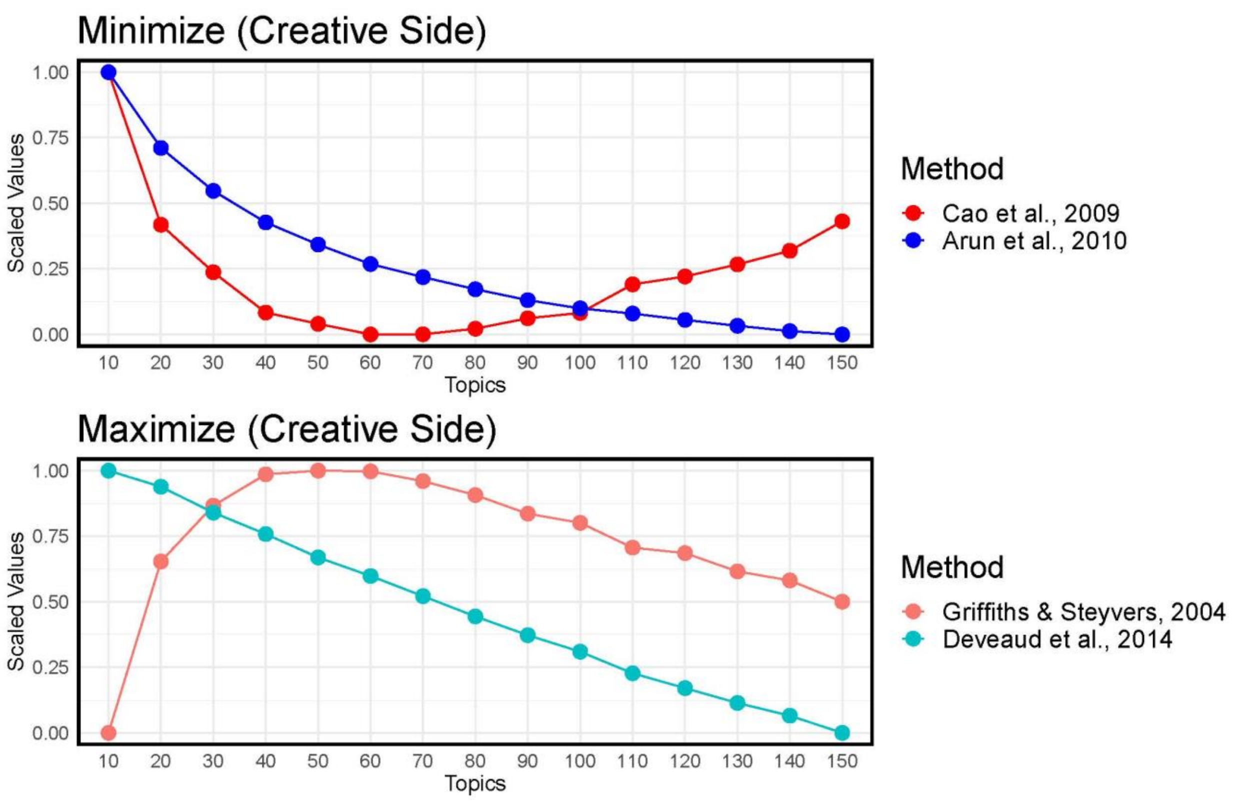

Table S1. Out-of-Sample Prediction Error for Prediction of Household Income by SAT Scores, Topics, and Dictionary Features Using 10-fold CV

Model	R^2
A. SAT Predicting Household Income	
SAT: Signif. Challenge	0.1171
SAT: Creative Side	0.1065
SAT EBRW: Signif. Challenge	0.0806
SAT EBRW: Creative Side	0.0739
SAT Math: Signif. Challenge	0.1175
SAT Math: Creative Side	0.1063
B. Topics Predicting Household Income	
Signif. Challenge	0.1050
Creative Side	0.0560
C. Dictionary Predicting Household Income	
Signif. Challenge	0.0973
Creative Side	0.0645

Table S2. Out-of-Sample Prediction Error for Prediction of SAT Scores by Topics and Dictionary Features using 10-fold CV

Model	R^2	RMSE
Topics		
Signif. Challenge	0.3039	142.75
Creative Side	0.2800	145.15
SAT EBRW: Signif. Challenge	0.2771	71.72
SAT EBRW: Creative Side	0.2429	73.63
SAT Math: Signif. Challenge	0.2734	85.95
SAT Math: Creative Side	0.2645	86.30
Dictionary		
Signif. Challenge	0.3019	142.95
Creative Side	0.3176	141.30
SAT EBRW: Signif. Challenge	0.2588	72.57
SAT EBRW: Creative Side	0.2660	72.42
SAT Math: Signif. Challenge	0.2766	85.71
SAT Math: Creative Side	0.2943	84.46

Table S3. Topics Generated from Merged Essays With Top Terms from Highest Probability and Frequent Exclusive Metrics

Topic (Merged Essays)	Highest Probability Words	Frequent Exclusive Words
Winning Competitions	competit, award, compet, win, nation, won, place	golf, compet, competit, won, award, decathlon, medal
Math	math, mathemat, subject, calculus, number, algebra, alway	math, algebra, bc, calculus, geometri, mathemat, trigonometri
AP Classes	cours, ap, school, take, honor, high, academ	cours, ap, placement, honor, enrol, rigor, advanc
Work and Goals	work, set, hard, apart, goal, believ, achiev	apart, set, california99, candid, hard, achiev, goal
Camping Swimming	run, camp, water, swim, race, cross, summer	swim, camper, swimmer, lifeguard, pool, cabin, polo
Social Anxiety	confid, speak, comfort, felt, feel, talk, fear	shi, comfort, afraid, zone, confid, fear, nervous
Gendered Activities	girl, boy, women, young, cheer, smile, name	girl, cheerlead, femal, stunt, women, guard, cheer
Fashion Style	color, black, cloth, wear, like, look, hair	makeup, outfit, dress, hair, wear, fashion, skin
Family Members	famili, parent, mother, father, brother, sister, home	brother, sister, mother, sibl, father, cousin, oldest
Medical Experiences	medic, hospit, doctor, bodi, patient, health, field	surgeon, physician, medic, kaiser, diabet, anatomi, nurs
Helping Others	peopl, help, can, make, way, differ, other	peopl, can, other, someon, everyon, differ, way
Despite Words	howev, one, may, rather, even, simpli, fact	simpli, rather, may, fact, truli, consid, howev
Latinx Family Issues	famili, educ, parent, school, immigr, mexico, live	undocu, latina, latino, los, angel, chicano, deport
Education Opportunity	colleg, educ, opportun9, take, advantag9, attend, school	advantag9, educ, colleg, opportun9, credit, graduat, prep
Classroom Experiences	class, teacher, test, student, studi, ask, question	test, teacher, exam, class, score, materi, review
Youth Volunteering	children, kid, volunt, help, teach, work, learn	children, disabl, kid, autism, volunt, center, buddi
Reading Writing	write, read, english, book, word, essay, stori	write, writer, essay, poem, poetri, literatur, reader
Making Planning	car, build, use, make, work, fix, drive	car, chess, wheel, bike, driver, cardboard, tape
Visual Art	creativ, art, express, draw, creat, artist, paint	draw, artwork, artist, art, ceram, canva, doodl
Travel	trip, travel, environment, environ, world, live, experi	island, aquarium, environment, itali, japan, rica, fish
Leadership Skills	skill, abl, develop, leadership, posit, allow, experi	skill, leadership, communic, develop, demonstr, abil, posit
Seeking Answers	question, book, like, research, read, answer, ask	telescop, astronom, map, probe, column, constel, encyclopedia
Mental Health	depress, mental, anxieti, bulli, drug, struggl, disord	alcohol, suicid, abus, bulli, gay, drug, harass
Outside School Programs	program, student, school, summer, mentor, academi, attend	upward, academi, mentor, bound, program, mente, workshop
Volunteer Cleaning	anim, clean, dog, trash, park, recycl, beach	hors, cadet, pet, dog, jrotc, trash, veterinari99
Work Experiences	store, custom, week, tabl, card, phone, two	bus, store, shop, cowork, card, custom, employe
Family Death	mom, dad, pass, felt, pain, cri, away	dad, mom, grandma, death, cri, die, grandpa
Motivations Goals	success, motiv, becom, goal, achiev, determin, continu	failur, persever, mindset, strive, capabl, motiv, success
Psychology Understanding	understand, other, friend, psycholog99, listen, situat, person	psycholog99, behavior, conflict, listen, mediat, empathi, disput

Group Leadership	club, member, presid, meet, join, offic, event	club, vice, secretari10, presid, copresid, nhs, rotari
Sports Experiences	team, teammat, captain, coach, season, practic, leader	captain, volleybal1010, teammat, team, varsiti, season, coach
World Histories	histori, world, learn, s, past, countri, event	histori, european, islam, histor, syria, egypt, war
China	chines, studi, student, also, time, china, school	china, provinc, hong, kong, chines, shanghai, wechat
Language Experiences	languag, english, spanish, learn, speak, cultur, understand	spanish, fluent, bilingu, french, eld, korean, languag
Cooking	food, cook, eat, make, meal, kitchen, bake	bake, recip, ingredi, culinari, chef, chees, cupcak
Civic Experiences	govern, polit, issu, elect, youth, confer, chang	attorney, voter, legisl, mayor, poll, civic, ballot
Time Management	time, work, help, get, school, abl, go	homework, manag, get, stress, done, stay, procrastin
Sensory Experiences	wall, hand, air, water, light, red, like	yellow, drip, nose, glass, fold, sun, stain
Sociocultural Diversity	cultur, differ, divers, world, peopl, american, societi	divers, asian, ethnic, racial, cultur, african, heritag
Business Economics	busi, econom, compani, market, product, manag, research	market, entrepreneur, entrepreneurship, econom, entrepreneuri, deca, ceo
Performance Art	perform, stage, act, show, audienc, play, charact	karat, theatr, theater, drama, actor, martial, actress
Computer Science	comput, scienc, program, code, technolog, game, learn	java, html, code, javascript, comput, python, hackathon
Photography	pictur, photographi, take, imag, photo, captur, camera	photographi, photograph, photo, pictur, captur, yearbook, imag
School Activities	student, school, event, high, leadership, campus, activ	asb, link, freshmen, homecom, crew, ralli, campus
Humor Storytelling	stori, charact, like, laugh, tell, joke, world	humor, tale, pun, potter, harri, superhero, joke
Group Assignments	group, project, work, idea, task, assign, member	group, assign, task, project, charg, present, partner
Work Money	job, money, pay, rais, work, parent, financi	money, buy, expens, $, dollar, sell, pay
Process Words	get, go, just, got, like, start, one	got, talk, bad, told, pretti, said, get
Boy Scouts	scout, ib, boy, project, troop, eagl, leader	scout, ib, troop, eagl, patrol, baccalaur, cub
Video Film	video, film, design, creat, media, edit, make	filmmak, film, editor, edit, footag, video, youtub
Family Church	church, youth, faith, god, cancer, grandmoth, grandfath	bibl, ministri, god, church, retreat, prayer, worship
Building Engines	engin, design, robot, build, project, work, use	robot, cad, aircraft, aerospac10, rocket, sensor, circuit
Human Nature	world, human, natur, passion, beyond, complex, explor	inher, manifest, notion, philosophi, nuanc, facet, myriad
Music	music, play, band, song, sing, piano, instrument	band, piano, guitar, drum, musician, violin, orchestra
Life Reflections	life, want, alway, never, know, love, can	everyth, anyth, never, happi, someth, els, ever
Time Cycles	day, hour, everi, night, week, time, morn	morn, night, sleep, wake, am, hour, pm
Life Challenges	life, challeng, face, live, situat, move, academ	life, adapt, situat, overcom, face, move, challeng
Sensory Responses	eye, word, moment, hand, began, head, back	stare, silenc, breath, mouth, utter, sigh, chest
HS Years	year, school, class, high, junior, freshman, sophomor10	junior, sophomor10, freshman, year, senior, high, class
Sports General	play, game, sport, player, soccer, basketbal1010, footbal	basebal, hockey, basketbal1010, soccer, tenni, softbal, refere

School Grades	grade, began, first, th, end, semest, improv	grade, semest, th, b, a, eighth, began
Dancing Art	danc, perform, dancer, movement, ballet, express, year	danc, dancer, ballet, choreograph, choreographi, polynesian1111, gymnast
Community Service	communiti11, help, servic, volunt, organ, event, local	homeless, donat, communiti11, servic, chariti, holiday, nonprofit
Preference Words	also, like, thing, realli, subject, lot, alway	realli, lot, thing, good, favorit, influenc, enjoy
Achievement Words	result, provid, initi, began, becam, academ, effort	dilig, remain, util, attain, endeavor, initi, simultan
Puzzles Problems	problem, solv, think, use, solut, find, way	solv, solut, problem, puzzl, logic, method, cube
Chemistry Biology	scienc, biolog, chemistri11, interest, research, subject, lab	chemic, biotechnolog1111, molecular, dna, molecul, biochemistri11, chemistri11
Tutoring Groups	help, tutor, colleg, avid, also, go, need	avid, tutor, ffa, et, ag, via, tutori
Physics	physic, world, understand, knowledg11, can, concept, univers	physic, newton, graviti, quantum, physicist, einstein, astronomi
New Experiences	new, learn, school, friend, even, first, found	new, found, friend, move, though, much, even

Table S4. Readability Scores Predicting Income and Total SAT Score, Merged Essays

Readability Metric	Adjusted R^2
A. Household Income	
Flesch Reading Ease	0.0158
Flesch-Kincaid Readability	0.0024
Dale-Chall	0.0180
Gunning Fog	0.0039
SMOG	0.0139
B. SAT Score	
Flesch Reading Ease	0.0778
Flesch-Kincaid Readability	0.0204
Dale-Chall	0.0924
Gunning Fog	0.0265
SMOG	0.0752

Table S5. Full Models Including Topics and Dictionary Features Predicting Income and SAT Score

Readability Metric	Adjusted R^2
A. Topics and Dictionary Predicting Household Income	
Merged	0.1746
Signif. Challenge	0.1231
Creative Side	0.0744
B. Topics and Dictionary Predicting SAT Score	
Merged	0.5256
Signif. Challenge	0.3605
Creative Side	0.3605

Table S6. SAT Score Mean and Standard Deviation by Income Decile, Merged Essays

Decile	Mean	σ
10 (highest)	1340.48	130.45
9	1306.40	141.03
8	1286.41	145.38
7	1260.13	149.10
6	1242.99	154.66
5	1201.95	162.14
4	1157.33	160.46
3	1128.28	163.14
2	1116.19	159.74
1 (lowest)	1101.86	157.85

Data S1.

Data and code can be found at https://dataverse.harvard.edu/dataverse/SAT_and_Essays. The data include SAT scores (composite, EBRW, and Math), household income, and variables generated from CTM and LIWC. The raw essays are not available in order to protect the privacy and anonymity of the applicants.

REFERENCES AND NOTES

1. J. Hyman, Act for all: The effect of mandatory college entrance exams on postsecondary attainment and choice. *Educ. Finance Policy* **12**, 281–311 (2017).

2. S. Goodman, Learning from the test: Raising selective college enrollment by providing information. *Rev. Econ. Stat.* **98**, 671–684 (2016).

3. D. Klasik, The act of enrollment: The college enrollment effects of state-required college entrance exam testing. *Educ. Res.* **42**, 151–160 (2013).

4. M. Hurwitz, J. Smith, S. Niu, J. Howell, The Maine question: How is 4-year college enrollment affected by mandatory college entrance exams? *Educ. Eval. Policy Anal.* **37**, 138–159 (2015).

5. E. J. Dixon-Román, H. T. Everson, J. J. McArdle, Race, poverty and SAT scores: Modeling the influences of family income on black and white high school students' SAT performance. *Teach. Coll. Rec.* **115**, 1–33 (2013).

6. R. Freedle, Correcting the SAT's ethnic and social-class bias: a method for reestimating SAT scores. *Harv. Educ. Rev.* **73**, 1–43 (2003).

7. M. Hout, Social and economic returns to college education in the United States. *Annu. Rev. Sociol.* **38**, 379–400 (2012).

8. C. Capuzzi Simon, "The test-optional surge," *New York Times*, 2015, vol. 12.

9. J. Furuta, Rationalization and student/school personhood in U.S. college admissions: The rise of test-optional policies, 1987 to 2015. *Sociol. Educ.* **90**, 236–254 (2017).

10. M. N. Bastedo, K. M. Glasener, K. C. Deane, N. A. Bowman, Contextualizing the SAT: Experimental evidence on college admission recommendations for low-SES applicants. *Educ. Policy* **2019**, 10.1177/0895904819874752 (2019).

11. M. N. Bastedo, N. A. Bowman, K. M. Glasener, J. L. Kelly, What are we talking about when we talk about holistic review? Selective college admissions and its effects on low-SES students. *J. Higher Educ.* **89**, 782–805 (2018).

12. M. L. Stevens, *Creating a Class* (Harvard Univ. Press, 2009).

13. D. M. Blei, J. D. Lafferty, Correlated topic models. *Adv. Neural Inf. Process. Syst.* **18**, 147–154 (2006).

14. M. E. Roberts, B. M. Stewart, D. Tingley, Stm: An R package for structural topic models. *J. Stat. Softw.* **91**, 1–40 (2019).

15. J. W. Pennebaker, R. L. Boyd, K. Jordan, K. Blackburn, "The development and psychometric properties of LIWC2015" (University of Texas at Austin, 2015).

16. S. Seraj, K. G. Blackburn, J. W. Pennebaker, Language left behind on social media exposes the emotional and cognitive costs of a romantic breakup. *Proc. Natl. Acad. Sci. U.S.A.* **118**, e2017154118 (2021).

17. L. Li, D. Demszky, P. Bromley, D. Jurafsky, Content analysis of textbooks via natural language processing: Findings on gender, race, and ethnicity in Texas U.S. history textbooks. *AERA Open* **6**, 10.1177/2332858420940312 (2020).

18. S. Munoz-Najar Galvez, R. Heiberger, D. McFarland, Paradigm wars revisited: A cartography of graduate research in the field of education (1980–2010). *Am. Educ. Res. J.* **57**, 612–652 (2020).

19. AJ Alvero, N. Arthurs, A. Lising Antonio, B. W. Domingue, B. Gebre-Medhin, S. Giebel, M. L. Stevens, AI and holistic review: informing human reading in college admissions, in *Proceedings of the AAAI/ACM Conference on AI, Ethics, and Society* (2020), pp. 200–206.

20. N. Arthurs, AJ Alvero, Whose truth is the 'ground truth'? College admissions essays and bias in word vector evaluation methods, in *Proceedings of the 13th International Conference on Educational Data Mining* (EDM, 2020).

21. J. W. Pennebaker, C. K. Chung, J. Frazee, G. M. Lavergne, D. I. Beaver, When small words foretell academic success: The case of college admissions essays. *PLOS ONE* **9**, e115844 (2014).

22. S. Jones, “Ensure that you stand out from the crowd”: A corpus-based analysis of personal statements according to applicants’ school type. *Comp. Educ. Rev.* **57**, 397–423 (2013).

23. S. Daenekindt, J. Huisman, Mapping the scattered field of research on higher education. A correlated topic model of 17,000 articles, 1991–2018. *High. Educ.* **80**, 571–587 (2020).

24. F. De Battisti, A. Ferrara, S. Salini, A decade of research in statistics: A topic model approach. *Scientometrics* **103**, 413–433 (2015).

25. D. Cutolo, S. Ferriani, G. Cattani, Tell me your story and I will tell your sales: A topic model analysis of narrative style and firm performance on Etsy, in *Aesthetics and Style in Strategy*, G. Cattani, S. Ferriani, F. Godart, S. V. Sgourev, Eds. (Emerald Publishing Limited, 2020). pp. 119–138.

26. J. W. Mohr, P. Bogdanov, Introduction—Topic models: What they are and why they matter. *Poetics* **41**, 545–569 (2013).

27. Y. R. Tausczik, J. W. Pennebaker, The psychological meaning of words: LIWC and computerized text analysis methods. *J. Lang. Soc. Psychol.* **29**, 24–54 (2010).

28. J. W. Pennebaker, M. E. Francis, R. J. Booth, *Linguistic Inquiry and Word Count: LIWC 2001* (Lawrence Erlbaum Associates, 2001).

29. R. Zwick, J. Greif Green, New perspectives on the correlation of SAT scores, high school grades, and socioeconomic factors. *J. Educ. Meas.* **44**, 23–45 (2007).

30. R. Flesch, A new readability yardstick. *J. Appl. Psychol.* **32**, 221–233 (1948).

31. E. Dale, J. S. Chall, A formula for predicting readability: Instructions. *Educ. Res. Bull.* **27**, 37–54 (1948).

32. G. H. McLaughlin, Smog grading—A new readability formula. *J. Read.* **12**, 639–646 (1969).

33. J. P. Kincaid, R. P. Fishburne Jr., R. L. Rogers, B. S. Chissom, "Derivation of new readability formulas (automated readability index, fog count and Flesch reading ease formula) for Navy enlisted personnel" (Naval Technical Training Command, Research Branch, 1975).

34. R. Gunning, *The Technique of Clear Writing* (McGraw-Hill, 1952).

35. D. Hossler, E. Chung, J. Kwon, J. Lucido, N. Bowman, M. Bastedo, A study of the use of nonacademic factors in holistic undergraduate admissions reviews. *J. Higher Educ.* **90**, 833–859 (2019).

36. K. O. Rosinger, K. S. Ford, J. Choi, The role of selective college admissions criteria in interrupting or reproducing racial and economic inequities. *J. Higher Educ.* **92**, 31–55 (2020).

37. J. Karabel, *The Chosen: The Hidden History of Admission and Exclusion at Harvard, Yale, and Princeton* (Houghton Mifflin Harcourt, 2006).

38. N. Lemann, *The Big Test: The Secret History of the American Meritocracy* (Macmillan, 2000).

39. N. Garg, H. Li, F. Monachou, Standardized tests and affirmative action: The role of bias and variance, in *Proceedings of the 2021 ACM Conference on Fairness, Accountability, and Transparency* (2021), pp. 261–261).

40. N. Schmitt, J. Keeney, F. L. Oswald, T. J. Pleskac, A. Q. Billington, R. Sinha, M. Zorzie, Prediction of 4-year college student performance using cognitive and noncognitive predictors and the impact on demographic status of admitted students. *J. Appl. Psychol.* **94**, 1479–1497 (2009).

41. Y. Attali, J. Burstein, Automated essay scoring with e-rater® V. 2. *J. Technol. Learning Assessment* **4** (2006); https://ejournals.bc.edu/index.php/jtla/article/view/1650.

42. F. Polli, J. Yoo, Systems and methods for data-driven identification of talent, U.S. Patent 16/013,784 (24 January 2019).

43. B. Bernstein, Elaborated and restricted codes: Their social origins and some consequences. *Am. Anthropol.* **66**, 55–69 (1964).

44. W. Labov, *Sociolinguistic Patterns* (University of Pennsylvania Press, 1973).

45. S. B. Heath, *Ways with Words: Language, Life and Work in Communities and Classrooms* (Cambridge Univ. Press, 1983).

46. P. J. Miller, D. E. Sperry, Déjà vu: The continuing misrecognition of low-income children's verbal abilities, in *Facing Social Class: How Societal Rank Influences Interaction*, S. T. Fiske, H. R. Markus, Eds. (Russell Sage Foundation, 2012), pp. 109–130.

47. B. Hart, T. R. Risley, The early catastrophe: The 30 million word gap by age 3. *American Educator* **27**, 4–9 (2003).

48. D. Nguyen, A. S. Doğruöz, C. P. Rosé, F. de Jong, Computational sociolinguistics: A survey. *Comput. Linguist.* **42**, 537–593 (2016).

49. D. Nguyen, M. Liakata, S. DeDeo, J. Eisenstein, D. Mimno, R. Tromble, J. Winters, How we do things with words: Analyzing text as social and cultural data. *Front. Artif. Intell.* **3**, 62 (2020).

50. D. T. Campbell, Assessing the impact of planned social change. *Eval. Prog. Plann.* **2**, 67–90 (1979).

51. K. Benoit, K. Watanabe, H. Wang, P. Nulty, A. Obeng, S. Müller, A. Matsuo, Quanteda: An R package for the quantitative analysis of textual data. *J. Open Source Softw.* **3**, 774 (2018).

52. M. F. Porter, "Snowball: A language for stemming algorithms" (2001).

53. N. Murzintcev, Select number of topics for LDA model (2016); https://cran.r-project.org/

web/packages/ldatuning/vignettes/topics.html.

54. T. L. Griffiths, M. Steyvers, Finding scientific topics. *Proc. Natl. Acad. Sci. U.S.A.* **101** (suppl. 1), 5228–5235 (2004).

55. R. Arun, V. Suresh, C. E. Veni Madhavan, M. N. Narasimha Murthy, On finding the natural number of topics with latent dirichlet allocation: Some observations, in *Pacific-Asia Conference on Knowledge Discovery and Data Mining* (Springer, 2010), pp. 391–402.

56. R. Deveaud, E. SanJuan, P. Bellot, Accurate and effective latent concept modeling for ad hoc information retrieval. *Document Nuḿerique* **17**, 61–84 (2014).

57. J. Cao, T. Xia, J. Li, Y. Zhang, S. Tang, A density-based method for adaptive LDA model selection. *Neurocomputing* **72**, 1775–1781 (2009).

58. T. Hastie, R. Tibshirani, J. Friedman, *The Elements of Statistical Learning: Data Mining, Inference, and Prediction* (Springer Science & Business Media, 2009).

59. E. M. Airoldi, J. M. Bischof, Improving and evaluating topic models and other models of text. *J. Am. Stat. Assoc.* **111**, 1381–1403 (2016).
